# Antimicrobial Peptides from Plants

**DOI:** 10.3390/ph8040711

**Published:** 2015-11-16

**Authors:** James P. Tam, Shujing Wang, Ka H. Wong, Wei Liang Tan

**Affiliations:** 1 School of Biological Sciences, Nanyang Technological University, Singapore, Singapore; E-Mails: wangshujing@mail.tsinghua.edu.cn (S.W.); hwka@ntu.edu.sg (K.H.W.); TANW0209@e.ntu.edu.sg (W.L.T.); 2 Department of Pharmacology and Pharmaceutical Sciences, School of Medicine, Tsinghua University, Beijing 100084, China

**Keywords:** plant antimicrobial peptides, cysteine-rich peptides, cystine knot, thionin, defensin, hevein, knottin

## Abstract

Plant antimicrobial peptides (AMPs) have evolved differently from AMPs from other life forms. They are generally rich in cysteine residues which form multiple disulfides. In turn, the disulfides cross-braced plant AMPs as cystine-rich peptides to confer them with extraordinary high chemical, thermal and proteolytic stability. The cystine-rich or commonly known as cysteine-rich peptides (CRPs) of plant AMPs are classified into families based on their sequence similarity, cysteine motifs that determine their distinctive disulfide bond patterns and tertiary structure fold. Cystine-rich plant AMP families include thionins, defensins, hevein-like peptides, knottin-type peptides (linear and cyclic), lipid transfer proteins, α-hairpinin and snakins family. In addition, there are AMPs which are rich in other amino acids. The ability of plant AMPs to organize into specific families with conserved structural folds that enable sequence variation of non-Cys residues encased in the same scaffold within a particular family to play multiple functions. Furthermore, the ability of plant AMPs to tolerate hypervariable sequences using a conserved scaffold provides diversity to recognize different targets by varying the sequence of the non-cysteine residues. These properties bode well for developing plant AMPs as potential therapeutics and for protection of crops through transgenic methods. This review provides an overview of the major families of plant AMPs, including their structures, functions, and putative mechanisms.

## 1. Introduction

Higher plants have a broad range of defense mechanisms to counter physical, chemical and biological stress such as drought, cold, heavy metal, pollutants and pathogen attacks from fungi, bacteria and viruses. In response to infection by a variety of pathogens, plants display up-regulation of a set of genes associated with systemic acquired resistance [1]. General resistance is accomplished by the release of secondary metabolites like phytoalexins, tannins and polyphenolic compounds, and the generation of pathogenesis-related (PR) proteins. PR proteins were first discovered in the early 1970s in tobacco leaves in response totobacco mosaic virus infections and were later defined as the induced proteins that are released during pathogenic attacks [2,3]. According to a recent review, there are at least 17 families that have been detected and isolated that possess a wide range of defense-related properties, including antibacterial, antifungal, antiviral, anti-oxidative activity, chitinase and proteinase inhibitory activities [1,2,3,4]. This review will focus on peptides which possess antimicrobial activity, namely thionin (PR-13 family), defensin (PR-12 family), hevein-like peptide, knottin, α-hairpinin, lipid transfer protein (PR-14 family) and snakin.

Antimicrobial peptides (AMPs) are ubiquitous and found as host defenses against pathogens and pests in diverse organisms ranging from microbes to animals [5]. AMPs exist in different molecular forms, although the majority of them are linear peptides from insects, animals, and plants. Nevertheless, bacteria produce polycyclic peptides such as lantibiotics, and all major forms of life produce circular peptides which include bacteriocins from bacteria, cyclotides from plants and theta-defensins from animals [6,7,8,9]. In plants, the majority of AMPs are Cys-rich [10], a feature that enables the formation of multiple disulfide bonds (usually two to six) that contribute to a compact structure and resistance to chemical and proteolytic degradation.

In general, plant AMPs share several common characteristics with those from microbes, insects and animals. They include features such as their molecular forms, positive charge and amphipathic nature, all of which are primarily related to their defensive role(s) as membrane-active antifungals, antibacterials, and antivirals. These features, in addition to being Cys-rich, are well represented by two plant AMP families, thionins and plant defensins. Other families of plant AMPs act on pathogens differently from animal AMPs. For example, hevein-like peptides bind chitins, knottin-type peptides inhibit enzymes such as proteases, and lipid transfer proteins bind lipids to disrupt microbial penetration into cell membranes.

Classification of plant AMP families is largely based on their Cys motifs which exhibit a characteristic Cys pattern with a defined number of non-Cys residues between the two neighboring Cys. Currently, the number of AMPs isolated from a limited number of plants already exceeds a thousand and is likely to increase in the future. Sequence analysis and genomic data mining using these Cys motifs have revealed that Cys-rich peptides (CRPs) with AMP characteristics are under-predicted [11]. In model plants, such as rice and *Arabidopsis*, CRPs may account for about 3% of the expressed proteins.

Like animal AMPs, AMP expression in plants is constitutive or induced and often tissue-specific. Moreover, plant AMPs are evolvable with hypervariable sequences encased in a particular scaffold characteristic of a given family of AMPs, which is analogous, but to a much lesser extent, to the molecular diversity of vertebrate immunoglobulin-based immunity.

This review aims to provide a general overview of the major families of plant AMPs, including their structures, functions, and putative mechanisms of defense. Additional details on plant AMPs can be found in a number of excellent previous reviews [12,13,14,15,16,17,18,19,20,21,22,23,24,25]. Furthermore, information on AMPs from diverse organisms can be accessed through several databases, such as APD, APD2 [26,27], YADAMP [28], DAMPD [29], and PhytAMP (specific for plant AMPs) [10].

## 2. Classification and Characteristics

Plant AMPs are divided into families based on their sequence similarity, Cys motifs, and distinctive disulfide bond patterns which, in turn, determine their tertiary structure folding. Table 1 lists the major families of plant AMPs based on these criteria; families include thionins, defensins, hevein-like peptides, knottin-type peptides (linear and cyclic), lipid transfer proteins, α-hairpinin families, snakins, and unclassified CRP-AMPs. In addition, non-CRP AMPs, which may be rich in other amino acids, are also described in this review, including the Gly-rich peptide (GRP) Pg-AMP1, the Gly- and His-rich peptide shepherins, and peptides of less than 10 amino acids (aa), such as Cn-AMP1 and Cr-ACP1. The following defining features are found in plant AMPs:

1. Mostly characterized as moderate-size (MW of 2–6 kDa), basic, CRPs with two to six intra-molecular disulfide bonds.
2. Members within a family are classified based on Cys motif, sequence similarity and are conserved in secondary and tertiary structure.
3. One or two additional disulfide bonds are found in members of thionins, defensins, and hevein-like peptides. These additional bonds bolster structural stability without affecting the general scaffold. Because the varying number of Cys residues can create confusion, we refer to AMPs within a family based on the number of Cys when necessary throughout this review (e.g., 6C-thionins contain six Cys and 8C-thionins have eight Cys).
4. In addition to being antimicrobial, AMPs also display “peptide promiscuity”, which refers to the multiple functions displayed by a single peptide.
5. All are ribosomally derived and bioprocessed from precursors, which often contain three domains: N- and C-terminal pro-domains and a mature AMP domain. Mature sequences are often hypervariable and display more variation than the conserved terminal domains in the preproprotein to give sequence diversity for adaptation.
6. Because of cross-bracing by multiple disulfide bonds, most CRP-AMPs with a molecular weight (MW) of 2–6 kDa are structurally compact with high thermal, chemical, and enzymatic stability.

**Table 1:** Major families of plant AMPs.

Peptide	S-S No.	Representative Member			Structural Motif
		Name	AA No.	Disulfide Motif	
6C-Thionin	3	Crambin	46	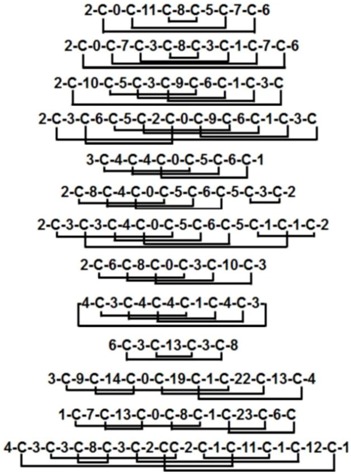	Gamma (Г) fold β1-α1-α2-β2-coil motif
8C-Thionin	4	β-Purothionin	45		
8C-Defensin	4	NaD1	47		CSαβ motif β1-coil-α-β2-β3
10C-Defensin	5	PhD1	47		
6C-Hevein	3	Ac-AMP1	29		Gly & Cys rich Central β strands & (short helical) side coils
8C-Hevein	4	Hevein	43		
10C-Hevein	5	EAFP1	41		
Knottin	3	PAFP-S	38		Cystine knot Short β strand & coil
Cyclic Knottin	3	Kalata B1	29		
α-Hairpinin	2	Ec-AMP1	37		α1-turn-α2
LTP	4	Maize LTP1	93		Hydrophobic cavity α1-α2-α3-α4-coil
LTP	4	Wheat LTP2	67		
Snakin *	6	Snakin-1	63		α -helices

* The disulfide and structural motif of Snakin-1 is predicted based on homology modelling.

### 2.1. Thionins

α-/β-Thionins are the prototypic plant AMP; they are cationic peptides of 45–48 aa with three or four disulfide bonds [12]. Initially, they were known as plant toxins because of their toxicity towards bacteria [30], fungi [31], plant and animal cells [32], as well as insect larvae [33]. The prototypic thionin with antimicrobial activity, α-purothionin, was isolated in the endosperm of wheat [30,34]. Following the discovery of α-purothionin, subsequent thionins isolated from other plants are labeled with descending letters of the Greek alphabet in the order of their discovery (e.g., α-thionins, β-thionins, and γ1/γ1-thionins).

**Figure 1:**
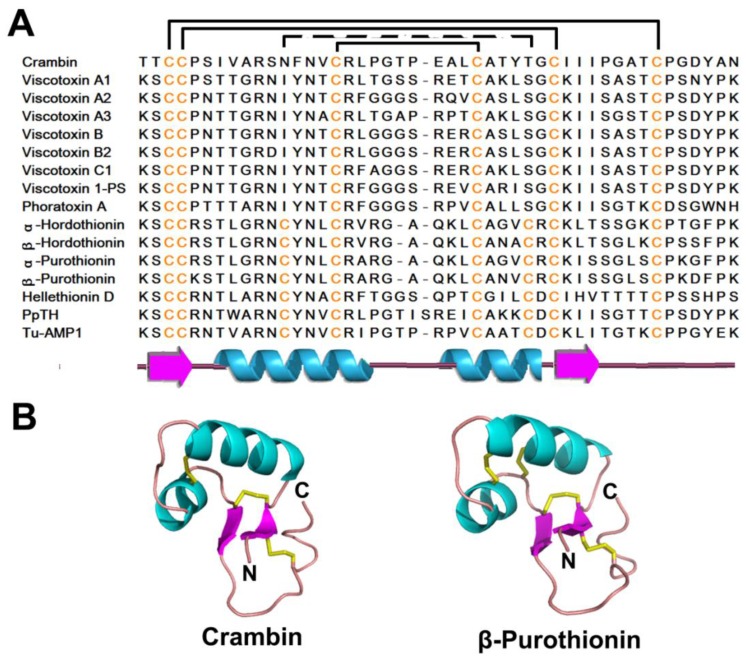
Sequences (**A**) and structures (**B**) of representative thionins. The secondary structure is represented by different colors: cyan-α helix; magenta-β strand; pink-random coil and yellow-disulfide bonds.

Classification of thionins is largely based on α-purothionin and includes the α-/β-thinoins of crambin, viscotoxins, phoratoxin A, hordothionins, and purothinoins. However, γ-thionins are considered part of the plant defensin family based on structural considerations. Thus, α-/β-thionins share a similar structural fold different from that of γ-thionins. For convenience, α/β-thionins with eight Cys residues will be hereafter referred to as 8C-thionins and those with six Cys designated 6C-thionins (Figure 1A,B).

#### 2.1.1. Occurrences, Distribution, and Biosynthesis

Thionins have been identified from monocots and dicots and are expressed in different tissues, such as seeds, leaves, and roots [35,36,37]. The expression of thionins can be induced by infection with various microbes [38,39] and has been shown to be related to the release of the hormone methyl jasmonate upon plant wounding or microorganism invasion [38,40,41]. Thionins are ribosomally derived and expressed as preproproteins, wherein the prothionin domain is flanked by two conserved sequences, the N-terminal signaling peptide and C-terminal acidic domain [35,38,42]. Mature thionin sequences display more variation than the conserved terminal domains in the preproprotein due to evolutionary pressure [43]. The three-domain precursor of plant AMPs in CRP families is typical and found in other CRP families, including defensins, hevein-like peptides, and knottin-type peptides.

#### 2.1.2. Structure

From the limited number of members identified thus far, thionins have relatively conserved amino acid sequences compared to other plant defense peptides (Figure 1A). They also share a conserved β1-α1-α2-β2-coil secondary structural motif, which forms a gamma (Г) fold, a special turn consists of three amino acid residues with the first and third residue connected by a hydrogen bond in the tertiary structure (Figure 1A,B).

**Figure 2:**
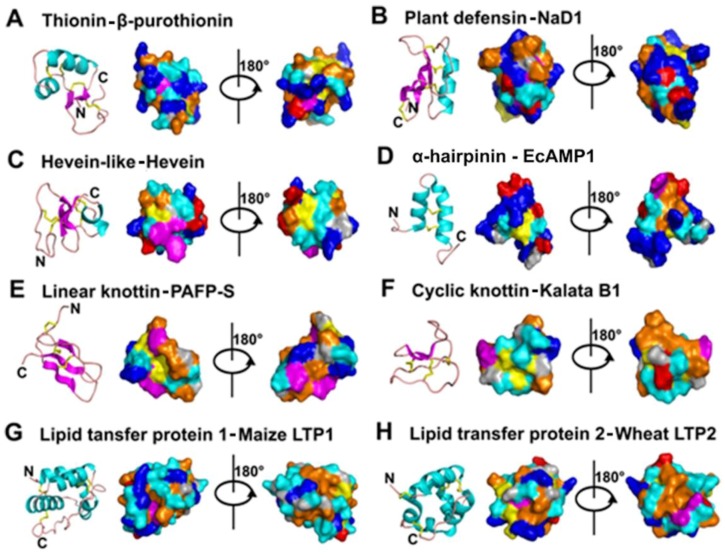
Cartoon illustration and surface plot of representative members of different antimicrobial peptides. The orientation of surface plot (left) of each representative member is the same as the cartoon illustration, while that of the right one is evolved from the left one by 180° turn around the y axis. Color representation rules for different amino acids are as follows: blue-positively charged (R, K & H), red-negatively charged (D & E), orange-hydrophobic (I, L, V, A, P & M), cyan-hydrophilic (S, T, E & Q), magenta-aromatic (F, Y & W), yellow-cysteine (C) and gray-glycine (G).

Thionins can be loosely classified as pseudocyclics because of an end-to-end disulfide bond linking the N- and C-termini, conferring a circular structural topology. They are, however, not true pseudocyclics because there are additional non-cysteine amino acids located at both the N- and C-termini. 8C-Thionins contain four conserved stabilizing disulfide bonds between CysI-CysVIII (Cys numbering in Roman numerals from N-to-C-termini) linking β1 to the C-terminal coil, CysII-CysVII linking the end of β1 with the beginning of β2, CysIII-CysVI linking α1 and the loop after α2, and CysIV-CysV linking α1 and α2. The 6C-thionins have the same Cys pairing as 8C-thionins except for the absence of the CysII-CysVII disulfide bond. The structure of 8C-thionins, including α-/β-purothionins, α-/β-hordothionin, and hellethionin D, are similar to that of 6C-thionins with the Г-shape but with minor differences in the C-terminal coil region [44,45,46]. The long arm of the Г fold comprises the α1-α2 region, and the short arm consists of β1 and the β2-coil. The large groove between the two arms is proposed to be important for the interaction between thionins and membrane lipids [43]. Most hydrophobic side chains cluster around the outer surface of the long arm, while the hydrophilic chains are located on the surface within the groove or outer face of the short arm (Figure 2A).

#### 2.1.3. Structure-Function Relationship

The 6C-thionins with three disulfide bonds include crambin, viscotoxins, and phoratoxin A. Most 6C-thionins are highly basic, amphipathic, and toxic, with the exception of crambin. Crambin is a neutral, hydrophobic, non-toxic peptide identified from *Crambe abyssinica* with two isomers, P22/L25 and S22/I25 [47,48]. High resolution structures of crambin have been determined by NMR and X-ray/neutron crystallography in both water and detergent [47,49,50,51,52,53]. Viscotoxins (including A1, A2, A3, B, B2, C1, and 1-PS) and phoratoxins from the mistletoes share a similar Г-shape with β1-α1-α2-β2-coil motif [54,55,56,57,58,59,60].

8C-Thionins with four disulfide bonds include α-/β-purothionins, α-/β-hordothionins, hellethionin-D, *Pyrularia Pubera* thionin (PpTH), and *Tulipa gensneriana* bulb-purified AMPs (Tu-AMPs). The monomeric conformation of the 45-aa α-hordothionin isolated from barley [61,62] was previously determined by NMR [62], while X-ray crystallography revealed a dimeric structure [61]. A study by Vila-Perello showed that removal of one disulfide bond from PpTH is sufficient to significantly alter its folding [63]. A 45% size-reduced form of PpTH was synthesized, which only contains residues 7–32 with the two antiparallel α-helices stabilized by two disulfide bonds. Size-reduced PpTH appeared to display the same antimicrobial activity and mechanism of action as intact PpTH in selected test microorganisms [64].

Tu-AMP1 and Tu-AMP2 are antibacterial, antifungal, and can reversibly bind chitin, a key constituent of the cell wall of fungi and exoskeletons of invertebrates, such as insects, anthropods, and nematodes. Initially, they were suggested to be thionin-like peptides, although Tu-AMP2 is a heterodimer of two chains joined by disulfide bonds [65]. However, it is worthwhile to point out that in our review of plant CRPs, the occurrence of heterodimer is exceedingly rare. In our view, it remains to be determined whether the heterodimeric formation occurs during the isolation process.

#### 2.1.4. Mechanism of Action

Thionins are hydrophobic and likely elicit their toxicity to bacteria, fungi, and animal and plant cells via membrane interactions with their hydrophobic residues or/and positive surface charge [12,13,18,66,67,68]. The proposed mechanism of toxicity is attributed to lysis of cell membranes, but it is still under investigation [30,39,68,69,70]. Stec proposed a structural model of the thionin-phospholipid interaction to explain the solubilization and lysis of cell membranes [43].

Thionins are known to directly interact with membrane lipids apart from protein receptors [67,71,72]. *Pyrularia* thionin from the nuts of *Pyrularia pubera* mediates the influx of Ca^2+^ during certain cellular responses, while Tyr iodination reduces its hemolysis, phospholipase A2 activation, and cytotoxicity [32]. Structure-function studies have demonstrated that Lys1 and Tyr13 in thionins are highly conserved and proposed to be crucial to their toxicity, with the exception of non-toxic, non-lytic crambin. Instead, crambin contains Thr1 and Phe13 residues [32,43,73,74]. Furthermore, Arg10 is suggested to be important to the folding stability of all thionins, as it is an abundant source of hydrogen bonds between β1, α1, and the C-terminal coil [75].

### 2.2. Plant Defensins

Plant defensins are the best known, and likely most abundant, of all plant AMPs with membranolytic functions, according to data mining of selected plant genomes. They are cationic peptides of 45–54 aa with four to five disulfide bonds [76]. Plant defensins have diverse biological functions which include antifungal [77,78,79,80,81], antibacterial [82,83], and α-amylase and trypsin inhibitory activity [84,85]. In addition to being antimicrobial, plant defensins are also involved in the biotic stress response, as well as plant growth and development.

Plant defensins were first identified as γ-thionins, γ1-hordothionin, and γ1-/γ2-purothionins from wheat and barley grains [86,87]. Thus, they were initially classified as γ-thionins due to their limited sequence identity (25%) with α-/β-thionins. Later, they were found to be unrelated to thionins based on structural features [88]. In 1995, they were grouped as plant defensins based on their sequence, structure, and function similarities with mammalian and insect defensins [76,78,88,89].

#### 2.2.1. Occurrences, Distribution, and Biosynthesis

Plant defensins include over 100 members from a wide range of plants, including wheat, barley, tobacco, radish, mustard, turnip, arabidopsis, potato, sorghum, soybean, cowpea, and spinach, among others [15,90]. They have been identified in multiple tissues, tubers [79,91], leaves [79], pods [92], and flowers [93,94,95], with the majority identified from seeds and roots [96]. Two types of precursors have been identified in plant defensins, wherein the dominant group is composed of the N-terminal signal peptide and a mature plant defensin domain [97], while the minor group is composed of an extra C-terminal acidic pro-domain of 33 aa reportedly associated with the vacuolar sorting mechanism since defensins with this domain were found in vacuoles and those without were found in the outer cell layers [16,79,95].

#### 2.2.2. Structures

**Figure 3:**
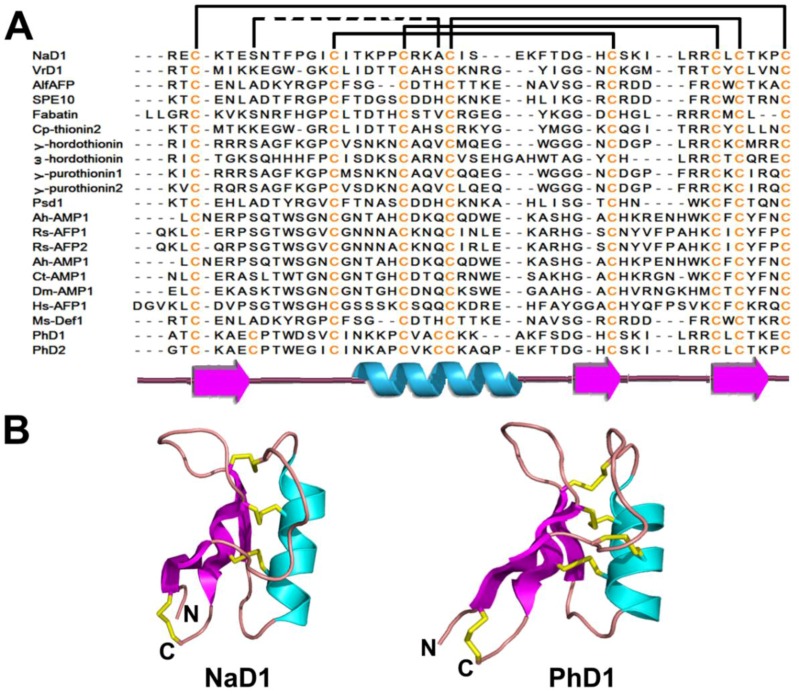
Sequences (**A**) and structures (**B**) of representative plant defensins. The secondary structure is represented by different colors: cyan-α helix; magenta-β strand; pink-random coil and yellow-disulfide bonds.

Plant defensins are generally characterized by four conserved disulfide bonds (except for PhDs, a 10C-plant defensin isolated from *Petunia hydrida*) with the outer disulfide pair as an end-to-end inner disulfide bridge and the inner three pairs of disulfide bonds forming a cystine knot (see knottins). A secondary structure characteristic of plant defensins is a Cys-stabilized αβ (CSαβ) motif of the cysteine knot. The CSαβ motif was first characterized in charybdotoxin, a K^+^ channel blocker isolated from scorpions [98] and named by Cornet *et al.* [99]. This motif forms a β1-coil-α-β2-β3 pattern in the secondary structure, where the α-helix is parallel to three antiparallel β-strands (Figure 3A,B). The CSαβ scaffold is stabilized and characterized by: (1) two disulfide bonds between the CXXXC motif from the α-helix and the CXC motif in the central β3 strand; (2) one disulfide bond between the β2 strand and the first loop before the α-helix; and (3) an end-to-end disulfide bond between the N- and C-termini, a disulfide connective similar to thionins. However, plant defensins (γ-thionins) differ greatly from α-/β-thinoins in their secondary and tertiary structures. In the secondary structure, α-/β-thionins show a typical β1-α1-α2-β2-coil motif, whereas γ-thionins display a β1-coil-α-β2-β3 motif. At the tertiary structural level, β1 and β2 orient antiparallel in α-/β-thinoins, whereas the corresponding β1and β3 in γ-thionins are oriented in parallel.

Similar to thionins, plant defensins are pseudocyclics (see thionins), with conservation of the disulfide bond between the first and last Cys, conferring a circular structural topology by connecting the side chains of the N- and C- termini.

Plant defensins have been reported to be stable in harsh conditions that mimic the digestive system and decoction process, including high temperature (~85 °C), low pH (~2.0), and oxidative and proteolytic environments [100,101,102]. Although defensins show conservation of the CSαβ motif, there are variations in the number of disulfide bonds, primary sequences, and tertiary structures [89,103,104]. It has been shown that Ala substitutions of non-Cys residues on VrD1 are tolerated in the CSαβ motif although the structural stability and inhibitory effects vary among the mutants [105].

#### 2.2.3. Structure-Function Relationship

Based on the number of Cys, plant defensins are further divided into 8C-plant defensins with four disulfides and 10C-plant defensins with five disulfide bonds.

The 8C-plant defensins include NaD1, VrD1, AlfAFP, ω-hordothionin, Cp-thionin II, Rs-AFPs, Psd1, Fabatins, and Ms-Def1 (Figure 3A,B). NaD1 (47 aa) was identified from the outer cell layers of different flower parts of the ornamental tobacco *Nicotiana alata*. Its distribution is consistent with its protective roles in reproductive organs. *In vitro* results showed that NaD1 inhibits the growth of plant pathogens *Botrytis cinerea* and *Fusarium oxysporus* [95,106]. VrD1 (46 aa) from the mung bean *Vigna radiata* not only inhibits protein synthesis, but is also antimicrobial and insect (bruchids)-resistant [107,108]. ω-Hordothionin (48 aa) purified from barley endosperm inhibits translational activity in both eukaryotic and prokaryotic cell-free systems [109]. The crystal structure of SPE10, identified from *Pachyrrhizus erosus* seeds [110], shows a dimer with each unit adopting the typical CSαβ motif [111]. Based on structural analysis and mutation studies, the dimeric conformation of SPE10 was suggested to be associated with its function, while the hydrophobic patch on the molecular head is necessary for its antifungal activity.

AlfAFP from *Medicago sativa* seeds is an antifungal peptide that provides robust resistance to the fungal pathogen *Verticillium dahliae* in transgenic potato plants [77,106]. Rs-AFPs (51 aa) purified from *Raphanus sativus* radish seeds are highly basic oligomeric proteins with an N-terminal pyroglutamic acid. They have a broad antifungal spectrum with an IC_50_ = 0.3–100 μg/mL [78,79,81]. Rs-AFPs are abundant in near-mature and mature seeds and released to create a fungal-suppressing microenvironment after disruption of the seed coat [79]. Ms-Def1 from *Medicago sativa* seeds strongly inhibits the fungal growth of *Fusarium graminearum in vitro* [112]; the inhibitory effect is reduced in the presence of Ca^2+^ ions. Homologous antifungal peptides were also reported from seeds of *Aesculus hippocastanum*, *Clitoria ternatea* (Ct-AMP1), *Dahlia merckii* (Dm-AMP1), *Lens culinaris* (Lc-def) and *Heuchera sanguine* (Hs-AFP1) [80,97,113].

Cp-thionin II (47 aa) was identified from *Vigna unguiculata* cowpea seeds and is antibacterial against both Gram-positive and Gram-negative bacteria such as *Staphylococcus aureus*, *Escherichia coli*, and *Pseudomonas syringae* with minimal inhibitory concentrations of 128, 64, and 42 μg/mL, respectively [90]. Psd1, a 46 aa peptide identified from seeds of the pea *Pisum sativum*, is antibacterial and acts as a K^+^ channel inhibitor based on a surface charge distribution analysis [114]. Fabatins isolated from the broad bean *Vicia faba* are active against both Gram-positive and Gram-negative bacteria, but inactive against the yeasts *Saccharomyces cerevisiae* and *Candida albicans* [115].

PhDs are 10C-plant defensins. From flowers of *Petunia* hybrids, Lay *et al.* reported two AMPs with antifungal activity, PhD1 (47 aa) and PhD2 (49 aa) [95]. A fifth disulfide bond exists between the α-helix and the loop after β1 within these defensins that do not alter the typical CSαβ topology [108]. The fifth disulfide bond appears only to change the corresponding hydrophobic interaction and hydrogen bond, as in 8C-plant defensins, to a covalent disulfide bond in PhD1 without altering the side-chain orientation of substituted residues.

The γ-thionins which have been reclassified as defensins, γ-hordothionin and ω-hordothionin from *Hordeum vulgare*, were shown to inhibit protein translation in rabbit reticulocytes and mouse liver extracts [87]. However, this inhibitory effect was not observed on plants, such as *Triticum aestivum*, *Cucumis sativus*, *Vicea sativa* and *H. vulgare*, and which may indicate a certain specificity in its mechanism of action. Similarly, no direct interaction was observed between the plant defensins and nucleic acids, which is the proposed mechanism of the inhibitory activity of protein synthesis by the plant thionin family [116]. Instead of protein translation, Mendez *et al.* suggested that ω-hordothionin may act on protein synthesis at the initiation and elongation step [109].

Defensins isolated from the seeds of *Sorghum bicolor*, S1α_1_, S1α_2_ and S1α_3_, were demonstrated to display inhibitory activity against α-amylase activity [84]. They are able to inhibit α-amylase obtained from the gut of the insects *Periplaneta americana* and *Locusta migratoria migratorioides*, and weakly inhibit the α-amylase from human saliva. Defensins from *Vigna unguiculata* and ω-hordothionin, from *H. vulgare*, also exhibit similar inhibitory activity against insect α-amylase [109,117,118], however, no significant inhibitory effect was observed on porcine α-amylase by plant defensins [119]. Several plant defensins exhibit the ability to inhibit ion channels. Kushmerick *et al.* showed that γ1-zeathionin and γ2-zeathionin from maize kernels block voltage-gated Na^+^ channels reversibly in intact mammalian GH3 cells using the patch-clamp technique [120]. These authors postulated that the ion channel inhibitory effect of defensins may be related to the similar 3D structure with scorpion neurotoxin, a well-known Na^+^ channel blocker. Plant defensins were also shown to inhibit Ca^2+^ channels [112]. However, no significant inhibitory effect was observed on K^+^ channels by plant defensins [119].

Two homologous peptides that display high sequence similarity to plant defensins were isolated from the plant *Cassia fistula*, denoted 5459 and 5144 according to their molecular weight [85]. The 5459 defensin was demonstrated to exhibit trypsin inhibitory activity, whereas no inhibitory effect was observed by the 5144 defensin. Another defensin peptide, Cp-thionin from *V. uguiculata* seeds, was reported to display inhibitory activity against pancreatic bovine trypsin [121].

An enzymatic activity was attributed to a plant defensin. Huang *et al.* cloned the defensin SPD1 from the roots of Ipomoea batatas and showed that it has the ability to regenerate dehydroascorbate (DHA) to ascorbic acid (AsA) in the presence of glutathione [122]. The peptide is also able to convert monodehydroascrobate (MDA) to AsA in the presence of NADH, functioning as a glutathione-dependent dehydroascorbate reductase. SPD1 is likely to function as a regulator of the redox state of AsA, which has been implicated in the response of the plant cell to reactive oxidative stress [123].

#### 2.2.4. Mechanism of Action

The structure-function relationship of plant defensins has been suggested to correlate with their positive charge and amphipathic nature, as illustrated by the structure-surface plot of NaD1 in Figure 2B. Thus, plant defensins could initially bind to microbial membranes through interactions with specific binding sites (“receptors”), as reported for Rs-AFP2, Hs-AFP1, and Dm-AMP1 [16,124,125]. Binding of plant defensins, such as Rs-AFP2 and Dm-AMP1, to the cell membrane results in the influx and efflux of positive ions like Ca^2+^ and K^+^ [126,127]. Lastly, Ms-Def1 is able to block the Ca^2+^ channel in a manner similar to the Ca^2+^ channel blocker KP4 [112]. Van der Weerden *et al.* demonstrated that NaD1 does not cause membranes permeabilization via a canonical mechanism which involves nonspecific insertion into membranes [128] but rather a cell wall dependent process, likely requiring a specific receptor. The mechanism of the fungicidal action of NaD1 is likely through permeabilization of the hyphae of *Fusarium oxysporum*, entering into the cytoplasm of the cell and inducing ROS oxidative stress [129]. Hayes *et al.* reported that the high-osmolarity glycerol (HOG) pathway is involved in the protection of the cell against NaD1 [130], indicating that the inhibition of the HOG pathway increases the activity of antimicrobial peptides against *Candida albicans*. Several reviews have discussed in detail the plant defensin mechanism of action [16,101,131,132].

### 2.3. Hevein-Like Peptides

Hevein-like peptides are basic peptides of 29–45 aa with three to five disulfide bonds. They are rich in Gly and contain conserved aromatic residues found in the hevein domain of lectins. Hevein domains bind to chitin [133,134,135], which is their primary target.

Hevein was first identified as the most abundant protein component from the latex of the rubber tree *Hevea brasiliensis* and displays strong antifungal activity *in vitro* [136,137]. It was also reported to be a major allergen from latex involved in human latex-fruit syndrome [138,139]. Similar to hevein, hevein-like peptides inhibit the growth of chitin-containing fungi and defend plants against attack from a wide range of fungal pathogens [133,140].

#### 2.3.1. Occurrences, Distribution and Biosynthesis

As a chitin-binding domain, the hevein domain is found in several plant lectins, natural variants of heveins (pseudo-hevein, wheat germ agglutinin, *Urtica dioica* agglutinin), and AMPs [134,141,142]. Similar to other families of CRP-AMPs, the hevein-like peptide is processed from a three-domain precursor. For example, the cDNA of the Ar-AMP precursor comprises a 25 aa N-terminal signal sequence, 30 aa mature peptide, and 34 aa C-terminal region which is cleaved during post-translational processing [143]. In 10C-hevein (hevein-like peptide containing 10 cysteine residues), there are two different precursor peptide structures in the C-terminal prodomain. The WAMP 10C-hevein from *Triticum kiharae* have precursor similar to other families CRP-AMPs, with a 45aa C-terminal region [144], while Ee-CBP 10C-hevein from *Euonymus europaeus* is produced as a chimeric precursor consisting of the mature peptide domain linked to a long C-terminal chitinase-like domain [145]. Andreev *et al.* [144] also showed that the WAMP-1 and WAMP-2 gene may have originated from ancestral chitinase genes and that a frame-shift deletion of the coding region for the catalytic domain led to the WAMP gene formation.

#### 2.3.2. Structure

**Figure 4:**
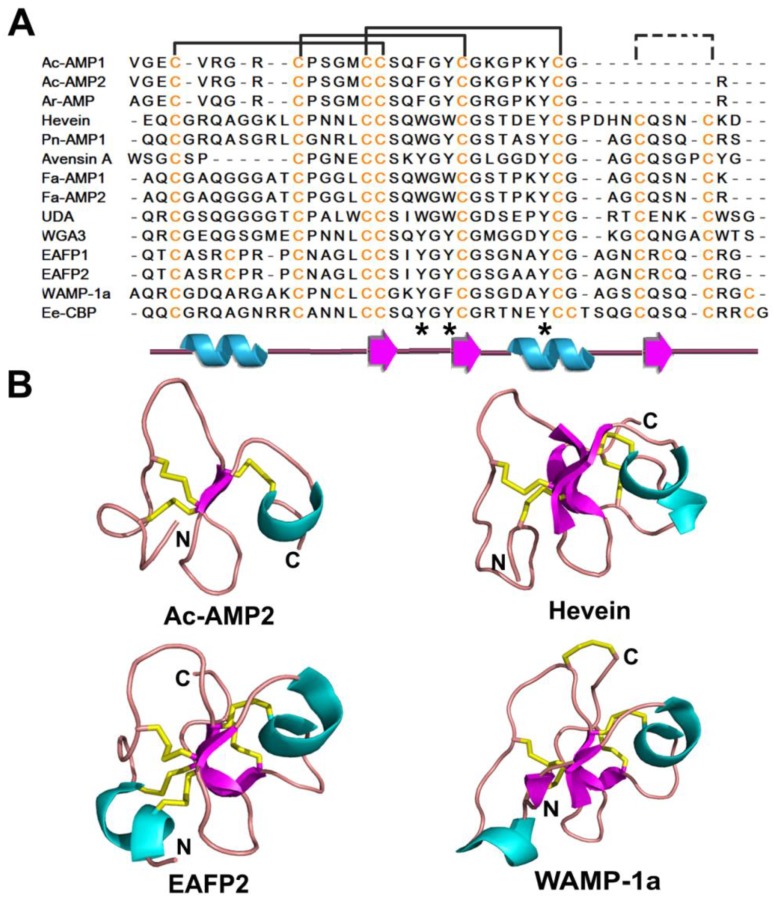
Sequences (**A**) and structures (**B**) of representative hevein-like peptides. The secondary structure is represented by different colors: cyan-α helix; magenta-β strand; pink-random coil and yellow-disulfide bonds.

Hevein-like peptides share conserved Cys, Gly, and several aromatic amino acid residues. They vary substantially in their primary sequences and number of disulfide bonds (from three to five; Figure 4A,B). Thus, hevein-like peptides can be divided into 6C-, 8C-, and 10C-hevein-like peptide subgroups based on the number of Cys they contain. All hevein-like peptides has a cysteine knot motif (see knottins). The solid-state and solution structures of the hevein domain, as determined by X-ray crystallography and NMR, respectively [142,146,147], provide the basis for analyzing the carbohydrate binding ability of this domain. Generally, hevein-like peptides contain a coil-β1-β2-coil-β3 secondary structural motif with variations based on the presence of short turns in the two long coils and β3 strand. Antiparallel β-strands form the central β-sheet of the hevein motif with the two long coils located on each side stabilized by disulfide bonds.

#### 2.3.3. Structure-Function Study

6C-Hevein-like peptides Ac-AMP1 and Ac-AMP2 isolated from *Amaranthus caudatus* seeds exhibit antimicrobial activity against both Gram-positive bacteria and plant pathogenic fungi with an IC_50_ 2–10 μg/mL [148]. Interestingly, their antimicrobial activity is antagonized by cations. Both Ac-AMP1 and Ac-AMP2 are similar to chitin-binding proteins and known to reversibly bind chitin in the C-terminal truncated fold of hevein [149]. This structure consists of a β-sheet with two antiparallel β-strands as the main central element, an N-terminal coil region, and C-terminal helical turn. The N-terminal coil region is linked to the two central β-strands by two disulfide bonds, while the C-terminal helical coil is connected to the first β-strand through a third disulfide bond. Ar-AMP (30 aa) purified from the seeds of amaranth (*Amaranthus retroflexus*) is another 6C-hevein-like peptide with antifungal activity [143].

Compared to Ac-AMPs, most hevein-like AMPs, including hevein, are 8C-hevein-like peptides with an additional C-terminal sequence containing the fourth disulfide bond. Thus, 8C-hevein contains a central β-sheet of three antiparallel β-stands, wherein the last β-strand is formed from the additional C-terminal sequences and oriented parallel to the fourth disulfide bond. The 6C-hevein32, a truncated form of hevein with the N-terminal 32 aa of hevein similar to Ac-AMP2, is defined as the minimum hevein domain since it presents comparable binding affinity for chito-oligosaccharides as native hevein [150]. Pn-AMP1 and Pn-AMP2 from seeds of the morning glory *Pharbitis nil* are highly basic (pI 12.02) and thermally stable. They exhibit potent antifungal activity against both chitin-containing and non-chitin-containing fungi with an IC_50_ 0.6–75 μg/mL, but lose their antifungal activity in acidic (pH 2.0) or reducing conditions [151]. As the first hevein-like peptides reported antifungal activity similar to thionins, Pn-AMPs have been successfully cloned into tomato and tobacco plants, endowing these transgenic plants with potent antifungal activities [152,153]. Fa-AMP1 and Fa-AMP2 from seeds of the buckwheat *Fagopyrum esculentum,* have antifungal and antibacterial activities with an IC_50_ of 11–36 μg/mL [82]. Avesin A from seeds of the oat *Avena sativa* represents another chitin-binding peptide with weak to moderate antifungal properties [154].

Several hevein-like AMPs contain five disulfide bonds (10C-hevein-like peptides), although the location of the fifth disulfide bond varies by peptide. For example, EAFP1 and EAFP2, each 41 aa long, were purified from the bark of the olive *Eucommia ulmoides Oliv* with an N-terminal pyroglutamic acid [155]. These peptides show inhibitory effects on both chitin-containing and chitin-free fungi with an IC_50_ 18–155 μg/mL and can be antagonized by Ca^2+^. Both solution and crystal structures indicate that EAFPs contain a chitin-binding domain similar to hevein-like peptides with a distinct Cys7-Cys37 fifth disulfide bond bridging the N-terminal coiled region with the third β-strand [156,157]. In contrast, the fifth disulfide bond within WAMP-1a isolated from the wheat *Triticum kiharae* connects the C-terminus to the central region of the structure [158,159]. Similarly, Ee-CBP from the bark of the spindle tree *Euonymus europaeus* is a potent antifungal peptide with an IC_50_ 1 μg/mL for the fungus *Botrytis cinerea* [160]. Ee-CBP has a primary sequence similar to other hevein-like peptides but with the fifth disulfide bond at the C-terminus.

#### 2.3.4. Mechanism of Action

As a chitin-binding domain, hevein is an excellent model for studying the carbohydrate-peptide interaction, which is reportedly mediated by hydrogen bonding and van der Waals forces. The carbohydrate-induced conformational change to the hevein domain is small based on NMR investigations of pseudo-hevein, a wheat germ agglutinin and truncated hevein mutant [142,161,162]. The interaction between the hydrophobic C-H groups of carbohydrates and the π-electron systems of aromatic amino acids (Trp21, Trp23, and Tyr30 in hevein; Figure 3A and Figure 8C) of hevein-like peptides appear to play an important role in chitin binding, as observed in Ac-AMP synthetic mutants, hevein, and a truncated form of hevein (hevein32) at key interacting positions [150,161,163,164]. Studies on Pn-AMPs showed that they rapidly penetrate fungal hyphae, leading to hyphal tip bursting, which disrupts the fungal membrane causing leakage of cytoplasmic materials [151].

In addition to the chitin binding function of hevein, Slavokhotova *et al.* showed an alternative function in which hevein plays a role in the plant defense against fungal infection [165]. WAMPs which contains an additional Ser at position 36 is able to inhibit the proteolytic activity of the secreted fungal protease fungalysin (Fv-cmp), a Zn-metalloproteinase, isolated from *Fusarium verticillioides*. This protease is able to truncate corn and Arabidopsis class IV chitinases by cleaving within the Gly-Cys site located in the chitin-binding domain of the plant chitinase. The presence of Ser36 prevents WAMP from being digested by Fv-cmp, allowing it to bind to fungalysin, and displace the plant chitinase, thus enabling the chitinase to remain intact and active [165].

### 2.4. Knottin-Type Peptides

Plant knottins belong to a superfamily, with members containing approximately 30 aa. They include inhibitors of α-amylase, trypsin and carboxypeptidase families as well as cyclotides. In general, they are among the smallest in size, but most diverse in functions of plant CRP-AMPs. Knottins typically comprise six Cys residues with conserved disulfide bonds between CysI-CysIV, CysII-CysV, and CysIII-CysVI, forming a cystine knot, but their Cys motifs differ among different subfamilies. Both plant defensins and hevein-like peptides also contain a cysteine-knot motif but they differ in their cysteine spacing.

One characteristic of this family is that they display a very broad range of bioactive functions which include hormone-like functions as well as enzyme-inhibitory, cytotoxic, antimicrobial, insecticidal, and anti-HIV activities [166]. Certain cystine-knot (CK) peptides with identical scaffold structures involved in multiple biological functions has been viewed as “peptide promiscuity” [167].

Historically, the knottin-type peptides were discovered as protease inhibitors sharing in common only in a cystine knot motif, and they are named collectively as cystine-knot inhibitor peptides, knottins. The prototypic knottin scaffold was first discovered in the subfamily of potato carboxypeptidase inhibitor (PCI) in 1982 [168]. The use of knottins also distinguishes the CK-CRPs from those initially described in the structures of the protein growth factors found in animals [169]. As a superfamily, they are believed to be the largest group of plant peptides associated with AMPs, surpassing defensins in the number of molecular forms and sequence diversity.

Knottins in the cyclotide and trypsin inhibitor families are found in two molecular forms, cyclic and linear, based on the presence or absence of backbone (head-to-tail) cyclization. In literature, cyclic knottins of the squash trypsin subfamily are often included in the cyclotide subfamily. Apart from their Cys residues, cyclic knottins and cyclotides share little sequence identity. Currently, both linear (acyclotides) and cyclic (cyclotides) forms of the cyclotide subfamily are found in plants.

#### 2.4.1. Occurrences, Distribution, and Biosynthesis

Linear knottins are found not only in plants, but also in other biological sources, including fungi, insects, and spiders. Thus, CK peptides with identical or related scaffold structures found in diverse life forms provide an example of parallel evolution of protein structures. Cyclotides and their acyclic variants are found only in plants, from the dicot plants of the *Rubiaceae*, *Violaceae*, *Cucurbitaceae*, *Fabaceae*, and *Solanaceae* families to a monocot plant of the *Poaceae* family, with predicted wide and abundant distribution [170,171,172,173,174,175,176,177].

Cyclotides and certain members of cyclic knottins of the squash family are produced from precursor proteins encoding one or more cyclotide domains. The precursor is composed of an endoplasmic reticulum signal region, pro-domain, one (or more) mature cyclotide domain(s), and a short C-terminal tail [172]. However, there are variations in their biosynthesis. A recent report on cyclotides such as cliotides (cT1-cT12) identified from *Clitoria ternatea* showed that they originate from chimeric precursors consisting of Albumin-1 chain A and cyclotide domains [173]. Studies have shown that an asparaginyl endoproteinase could be involved in the backbone cyclization of cyclotides [178,179,180,181]. Our laboratory has isolated one of the bioprocessing enzymes responsible for the backbone cyclization process from *C. ternatea*, butelase 1 [181]. Butelase 1 acts as a transamidase, cyclase and ligase and is C-terminal specific to produce Asx-Xaa bonds, with Xaa being a diverse group of residues. Butelase 1 cyclizes various peptides of plant and animal origin efficiently and is the fastest peptide ligase known. Linear variants of cyclotides share high sequence identity and contain a similar knottin scaffold but are biosynthetically unable to cyclize from their precursors [173,182]. Violacin A, a naturally occurring linear cyclotide from *Viola odorata*, lacks the essential bioprocessing signal, the C-terminal Asn residue required for cyclization due to the presence of a stop codon earlier in the C-terminal sequence [177].

Cystine knot α-amylase inhibitors (CKAIs) are plant-derived α-amylase inhibitors originally isolated from *Amaranthus hypocondriacus* [183]. They are the smallest family of proteinacous α-amylase inhibitors among the seven known families [184]. Unlike other knottins, these peptides are rich in proline residues, with at least one of them existing in a *cis-* configuration [185]. In recent studies, Nguyen *et al.* have isolated an additional three members of CKAIs from the leaves and flowers of *Wrightia religiosa* [186] and another five members from the leaves of *Allamanda cathartica* [187]. These CKAIs contain 30 residues, two residues shorter than AAI, and share high sequence homology to each other.

#### 2.4.2. Structure

A common knottin structural motif was initially defined in 1994 as a CK and triple-stranded β-sheet with a long loop connecting the first and second β-strand [166]. The first two disulfide bonds (between CysI-CysIV and CysII-CysV), together with their connecting backbone, form an embedded ring that is penetrated by the third disulfide bond (between CysIII-CysVI). Studies on the subfamily of squash trypsin inhibitors and PCIs showed that only two disulfide bonds (between CysII-CysV and CysIII-CysVI) in the knottin scaffold are highly conserved and sufficient to maintain the Cys-stabilized β-sheet motif [188,189]. It is worthwhile to point out that cystine-knot motifs appear to be common occurrence in plant CRP-AMPs. They are found, at the primary structure level, in plant defensins and heveins. However, they differ in the secondary and tertiary structure levels.

**Figure 5:**
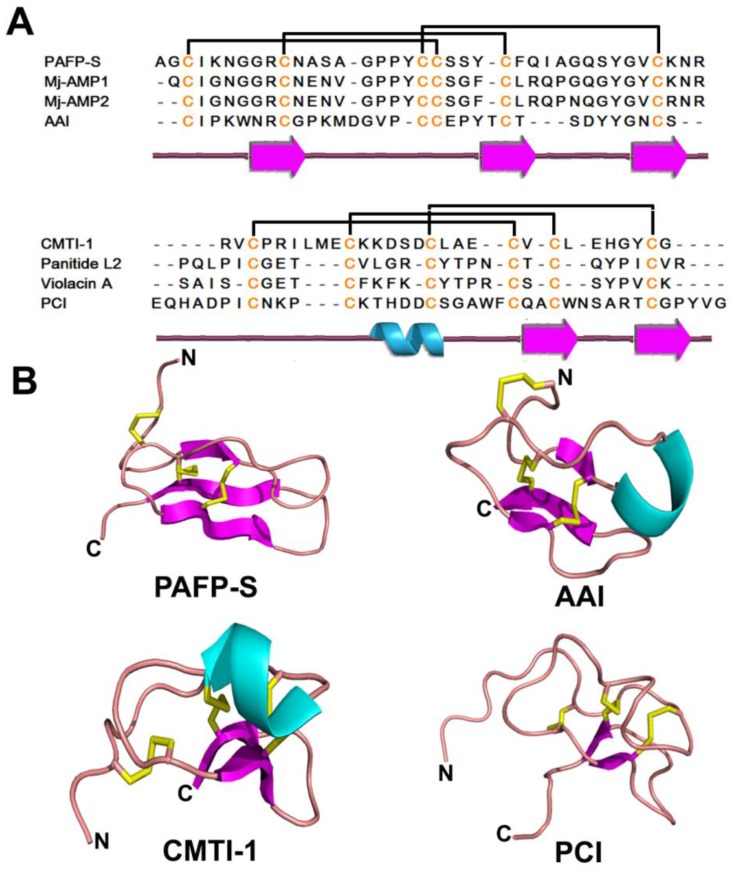
Sequences (**A**) and structures (**B**) of representative linear knottin-type peptides. The secondary structure is represented by different colors: cyan-α helix; magenta-β strand; pink-random coil and yellow-disulfide bonds.

Despite the common knottin motif, knottin-type peptides have hypervariable sequences, differing by their amino acid sequences, the length between CysIII-CysIV and CysIV-CysV, and the linear and cyclic nature of the peptide backbone (Figure 4 and Figure 5). Owing to the high sequence tolerance of the knottin scaffold and its diverse biological functions, the knottin scaffold has been used as a template for drug design. Knottins engineered by substitution of individual or several consecutive amino acids and/or insertion of additional amino acids without changing the structural integrity have been shown to provide novel bioactivity or increase stability [190].

Plant knottin-type peptides, particularly the subfamily of cyclotides, have been reported to possess high thermal, chemical, and enzymatic stability [186,191,192]. Cyclotides are also resistant to gastrointestinal proteases like trypsin, chymotrypsin, pepsin, or elastase [191,193], and certain members of cyclotide family can even penetrate the intestinal mucosa excised from rats [194,195]. Disulfide bonds in the knottin scaffold are crucial to its chemical and enzymatic stability based on studies of various members of cyclotides such as violacin A, kalata B1, and kalata B2 [177,191], whereas the cyclized backbone is important for exopeptidase resistance. For example, stability tests show that acyclic vilacin A is resistant to endopeptidase, trypsin, and thermolysin, comparable to cyclotides. The exoprotease aminopeptidase M cleaves the first two N-terminal residues of violacin A but leaves the third and fourth residues intact due to their proximity to the disulfide bond. Although no large differences are observed in the structure and flexibility between cyclotides and their corresponding linear analogs under standard conditions, simulation studies of linear and circular squash inhibitors revealed that cyclization increases resistance to high temperatures by limiting structure unfolding [192].

CKAIs exhibit another method in which these peptides are able to maintain stability against exopeptidases without a cyclic backbone structure, a pseudocyclic structure [186]. In wrightides, CKAIs isolated from the plant *W. religiosa*, the N-terminus and C-terminus are protected by the formation of disulfide bonds at the ultimate or penultimate residues. Structural analysis showed that this arrangement allows the termini to loop back to the peptide chain via the disulfide bonds especially at the N-terminal, forming a pseudocyclic structure. Allotides, CKAIs from *A. cathartica*, and AAI also exhibit similar structural features [183,187].

#### 2.4.3. Structure-Function Relationship

With the exception of the cyclotide subfamily, the majority of knottins, are linear. They include the subfamilies of PAFP-S, Mj-AMPs, insect α-amylase inhibitor, squash trypsin inhibitor CMTI-1 and carboxypeptidase A inhibitor, as well as several linear homology analogs of cyclotides (violacin A and panitide L2; Figure 5A,B). Several knottin-type peptides were identified as plant AMPs, such as PAFP-S, Mj-AMPs, and Psacotheasin (Ps). PAFP-S was identified from seeds of *Phytolacca americana* and models the typical knottin structure of antifungal peptides [196]. Mj-AMP1 and Mj-AMP2 extracted from *Mirabilis jalapa* seeds have a broad spectrum of antimicrobial activity, being active against all 13 tested fungal pathogens and two tested Gram-positive bacteria but inactive against Gram-negative bacteria and cultured human cells. Reduced and non-reduced SDS-PAGE results suggest that Mj-AMP1 and Mj-AMP2 exist as dimers in their native form [197]. Ps from *Psacothea hilaris* is a 34 aa antibacterial peptide with a minimal inhibitory concentration of 12.5–25 μM [198].

Knottin-type peptides act as α-amylase or protease (carboxypeptidase A or trypsin) inhibitors (Figure 5A,B) and propagate plant defense mechanisms by conferring resistance to insects, pests, and pathogens [199]. The α-amylase inhibitor identified from *A. cathartica* and *W. religiosa* are the smallest peptide inhibitor of α-amylase activity (30 aa) [186,187]. Wr-AI1 and Wr-AI2, CKAIs from the plant *W. religiosa*, have been demonstrated to inhibit the α-amylase activity isolated from *Tenebrio molitor* (yellow mealworm), but no inhibitory was observed in fungal or mammalian α-amylase. Similar α-amylase inhibitory activity was observed from allotide Ac4. However, it was found that allotides interact with TMA differently from AAI and wrightides due to variation in the N-terminal sequences and high content of *cis*-proline. AAI confers pest resistance to plants by targeting insect α-amylase, but does not interfere with α-amylase from mammalian digestive systems, suggesting that amaranth seed AAI could be an attractive candidate to endow pest resistance to transgenic plants.

Squash trypsin inhibitors are 27–32 aa knottin-type peptides (e.g., CMTI-II of both pumpkin *Cucurbita maxima* and fig leaf gourd *Cucurbita ficifolia* seeds) [200]. The first squash inhibitor containing the knottin scaffold was reported from the seeds from squash of the *Cucurbitaceae* family [201]. Most squash trypsin inhibitors have linear backbones, except for MCoTI-I and MCoTI-II. However, not all the protease inhibitor peptides are knottin-type, such as sun flower trypsin inhibitor, a 14 aa cyclic peptide braced by a central disulfide bond [202]. Another type of knottin with protease inhibitory function includes carboxypeptidase inhibitors from potatoes [145] and tomatoes [203]. Potato carboxypeptidase inhibitor (PCI) (39 aa) has long loops instead of the typical β-strands observed in other knottin-type peptides. It binds the active site of carboxypeptidase A with the C-terminal tail as an active fragment binding to protease in a stopper-like manner and uses some aromatic residues as secondary binding sites [204,205].

**Figure 6:**
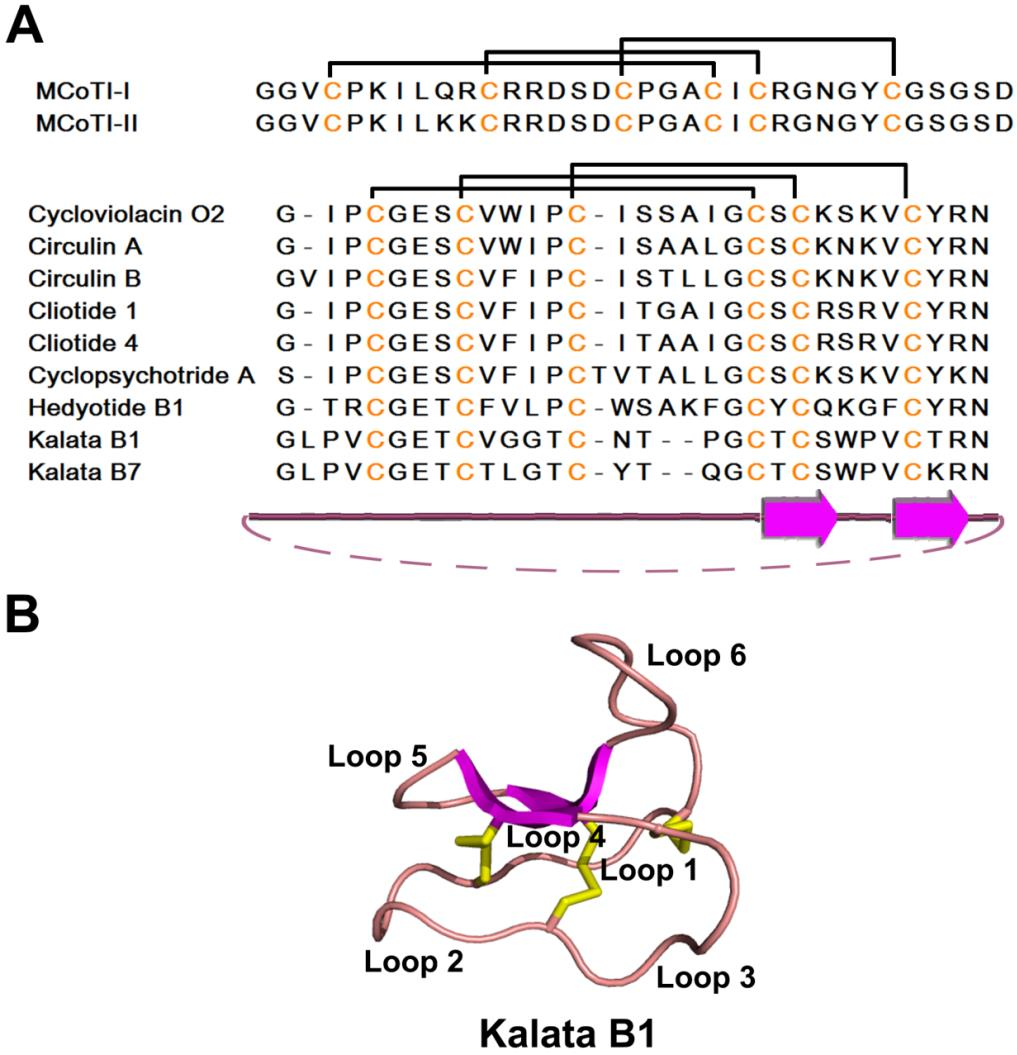
Sequences (**A**) and structures (**B**) of representative cyclic knottin-type peptides. The secondary structure is represented by different colors: magenta-β strand; pink-random coil and yellow-disulfide bonds.

A few linear variants of cyclotides have also been identified from the monocot rice plant *Panicum laxum* of the *Poaceae* family as Panitide L1-12 [175]. Several Panitides are active against *Escherichia coli* and cytotoxic to HeLa cells. The other subgroup of knottin-type peptides is cyclotides, including typical cyclotides and cyclic knotttins such as MCoTIs (Figure 6A,B). Cyclotides are 29–37 aa in length with a CK arrangement of three disulfide bonds which are widely involved in plant defense, as deduced from their activity against insects, nematodes, and mollusks [206,207,208]. The first cyclotide was identified in 1973 as an uterotonic agent from the African plant *Oldenlandia affinis*, a main component of medicinal tea used to accelerate childbirth [209]. Cyclotides have potential pharmacological functions, considering their antimicrobial, anti-HIV, anti-tumor, and neurotensin activities [22]. Cyclotides are found mainly in *Rubiaceae* (coffee), Violaceae (violet) and families. They are highly variable in sequence, but conserved in structure, and are divided into two types: Möbius and bracelet. Möbius types contain one *cis*-Pro in loop 5 and a twist in the cyclic backbone, while the bracelet type does not [210]. Yet, both types do not significantly differ from one another in the general scaffold structure.

Antimicrobial activity of cyclotides was first reported for four synthetically produced cyclotides isolated from coffee plants, kalata B1, circulin A, circulin B, and cyclopsychotride [211]. Subsequently, more cyclotides were reported to have antimicrobial activity. Cyclotides kalata B1 and B7 (29 aa each) from the tropical plant *Oldenlandia affinis* display antibiotic effects both in salt and salt-free medium [212]. While the 30 aa cycloviolacin O2 from *Viola odorata* is resistant to Gram-negative bacteria [213], cliotides cT1 and cT4 from *Clitoria ternatea* show antimicrobial activity against Gram-negative bacteria and cytotoxicity to HeLa cells [173]. Hedyotide B1, on the other hand, is a positively charged bracelet cyclotide from *Hedyotis biflora*, rich in aromatic residues and active against both Gram-positive and Gram-negative bacteria [214].

MCoTI-I and MCoTI-II are two squash trypsin inhibitors from *Momordica cochinchinensis* that contain a cyclized backbone [215] and have large sequences differences from typical cyclotides, as discussed above (Figure 6A). The C- to N-cyclization in MCoTI-II has no significant impact on the protein structure, though cyclized squash inhibitors are postulated to be less sensitive to exopeptidases [216]. Based on structural studies of MCoTI-II in free and complex forms, Heitz revealed that the cyclization and active site loops of MCoTI-II are flexible in solution, but converge into a single, well-defined conformation upon binding trypsin [217]. Compared to previously defined cyclotides, such as kalata B1 or circulin A, MCoTI-I and MCoTI-II share similar motifs wherein two disulfide bonds stabilize the β-sheet but differ greatly in their amino acid sequences and in loops 3 and 6 [217]. Furthermore, MCoTIs have an entirely charged surface *versus* the amphipathic nature of circulin A. These differences may explain functional disparities such as squash inhibitor MCoTIs having no antibacterial activity, unlike circulins and kalata B1.

#### 2.4.4. Mechanism of Action

Generally, knottin-type peptides with membranolytic functions are amphipathic in nature like other AMPs, a characteristic necessary for membrane interactions which implement their antimicrobial effects. For example, the surface plots of PAFP-S and kalata B1 show hydrophobic patches surrounded by several hydrophilic residues (Figure 2E,F). However, in contrast the strongly cationic-charged thionins and plant defensins, most cyclotides are unlikely to have a strong electrostatic interaction with membranes since they are normally weakly positive or neutral at physiological pH (Figure 2F) [218]. The interaction of cyclotides with membranes has been previously investigated *in vitro* using the detergent dodecylphosphocholine [219]. In this study, the structure of kalata B1 was not significantly altered upon binding the detergent; binding was largely mediated by the strong hydrophobic interactions between cyclotide loops and lipid tails of the detergent, as well as favored by the weak interactions between positively charged kalata B1 and the polar head of the detergent However, similar studies on kalata B2 and cycloviolacin O2 suggest that different cyclotides have different membrane binding modalities because of the varied location of hydrophobic patches in cyclotides [220].

#### 2.4.5. Knottin Scaffold in Pharmaceutical Engineering

The knottin scaffold is an excellent candidate for peptide-based pharmaceutical engineering [221] as a result of several features: (1) remarkable proteolytic, thermal, and chemical stability due to the CK and backbone cyclization of cyclotides; (2) feasibility of chemical synthesis due to small size; and (3) excellent sequence tolerance due to sequence variation in loop regions.

Synthesis of cyclotides and cyclic knottins. To exploit cyclotides and cyclic knottins for agricultural and medical use, it is necessary to develop efficient methods for their production. Methods employed to date include solid phase peptide synthesis [9,211,222,223,224,225,226,227], as well as chemo-enzymatic and biological methods using modified inteins [210,211,212,213] and Asn-endoprotease such as butelase-1 [228,229,230,231].

Irrespective of the means of its preparation, a linear precursor containing a macrocycle sequence is first generated, followed by a macrocyclizaion step to give a head-to-tail backbone-cyclized compound. The head-to-tail cyclization poses a substantial hurdle and challenge because it is highly entropy-disfavored due to the great distance between the N- and C-termini, but has been solved elegantly by the discovery of the thia zip cyclization reaction in 1997 [232]. Thia zip cyclization is an entropic reaction, characterized by a series of entropic ring expansion by making use of the multiple Cys residues in a CRP-linear precursor functionalized with an N-terminal Cys and a C-terminal thioester. This construct enables a thiol-thioester to form a thiolactone and then thiol-thiolactone exchange reactions in tandem, ending with a head-to-tail thiolactone which spontaneously forms a peptide bond through an S, N-acyl shift. In 1997, the first report on both Möbius and bracelet cyclotides have been synthesized by the thia zip reaction and regio-selective disulfide bond formation to guarantee the correct knottin scaffold [225]. Since then, many successful syntheses of cyclic CRPs based on thia zip cyclization have been reported. We have recently reviewed the progress of macrocyclization relevant to cyclotides and cyclic knottins [9]. Since 2012, new advances based on amide-to-amide transpeptidation reaction have made preparation of cyclotides, cyclic knottins and other macrocycles possible through chemical or biological means [181].

### 2.5. α-Hairpinin Family

**Figure 7:**
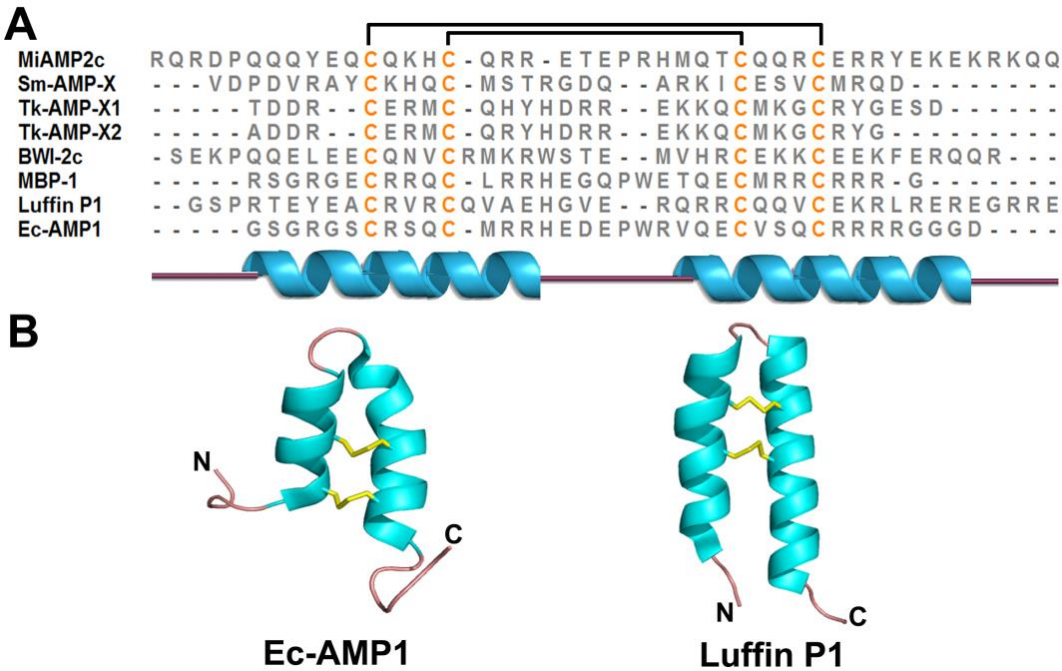
Sequences (**A**) and structures (**B**) of representative α-hairpinins. The secondary structure is represented by different colors: cyan-α helix; pink-random coil and yellow-disulfide bonds.

The α-hairpinin family is composed of Lys/Arg-rich plant defense peptides. α-Hairpinin AMPs share a characteristic C1XXXC2-(X)n-C3XXXC4 motif in their primary sequence and, more importantly, a helix-loop-helix secondary structure (Figure 7A,B). The helix-loop-helix or α1-turn-α2 motif has both α-helices oriented antiparallel and is stabilized by two disulfide bonds in the tertiary structure. This structure is a surprise finding in plant CRPs, and the α-hairpinin family is structurally distinguished from the β-strand decorated CRP-AMPs, such as thionins, defensins, and knottin-type peptides.

Thus far, only a limited number of α-hairpinin AMPs have been reported, including MBP-1, MiAMP2s, Ec-AMP1, Luffin P1, VhT1, BWI-2c, Tk-AMP-Xs, and Sm-AMP-X. MBP-1, a 33 aa peptide isolated from the maize kernel, inhibits spore germination and hyphal enlongation of several plant pathogenic fungi and bacteria *in vitro* [233]. MiAMP2 peptides (50 aa) isolated from the nut kernel of *Macadamia integrifolia* inhibit various plant pathogenic fungi *in vitro* [234]. They are produced from a 666 aa precursor protein homologous to vicilin 7S globulin. Ec-AMP1 from the seeds of the baryard grass *Echinochloa crus-gali* was reported to have antifungal activity against several phytopathogenic fungi with an IC_50_ = 1–10 μM. A confocal microscopy study showed that Ec-AMP1 binds the fungal conidia surface and then internalizes and accumulates in the cytoplasm without disturbing membrane integrity [235].

Tk-AMP-X1 and Tk-AMP-X2 extracted from the wheat *Triticum kiharae* and Sm-AMP-X from seeds of the chickweed *Stellaria media* are another two α-hairpinin members with antifungal activity [236,237]. Both are produced from multimodular precursor proteins. The Tk-AMP-X-related sequences have been shown to be widespread in crops such as barley, rice, and maize, which suggest the importance of this type of plant defense peptide. VhT1 from the seeds of *Veronica hederifolia* and BWI-2c from seeds of the buckwheat *Fagopyrum esculentum* represent a new family of trypsin inhibitors with an α-hairpinin structure and act as defensive peptides in plants [238,239]. Luffin P1, extracted from the seeds of the sponge gourd *Luffa cylindrical*, has been shown to have anti-HIV-1 activity in HIV-1-infected C8166 T-cell lines *in vitro* [240]. This study proposed that Luffin P1 displays a novel inhibitory mechanism owing to its charge complementation with viral and cellular proteins.

### 2.6. Lipid Transfer Proteins

Plant lipid transfer proteins (LTPs) and snakins (described in the following section) are two families of CRP-AMPs with MW >7 kDa, and are considered proteins. LTPs are cationic proteins of approximately 70 and 90 aa with eight Cys residues. They are distinguished from other CRP-AMPs by their lipid transfer activity, in which they bind a wide range of lipids including fatty acids (C10–C14), phospholipids, prostaglandin B2, lyso-derivatives, and acyl-coenzyme A. Consequently, they are also called non-specific LTPs [4,241,242]. LTPs can inhibit growth of fungus and some bacterial pathogens and are involved in the plant defense system. LTPs are subdivided into LTP1s (MW = 9 kDa) and LTP2s (MW = 7 kDa) based on their molecular mass.

#### 2.6.1. Occurrences, Distribution, and Biosynthesis

Plant LTPs have been identified in various species, such as seeds of the radish, barley, maize, *Arabidopsis*, spinach, grapevine, wheat, and onion [4,241,242,243,244,245,246]. They are synthesized as precursors containing a signal peptide of 20–25 aa and a mature protein with eight Cys [247].

#### 2.6.2. Structure

**Figure 8:**
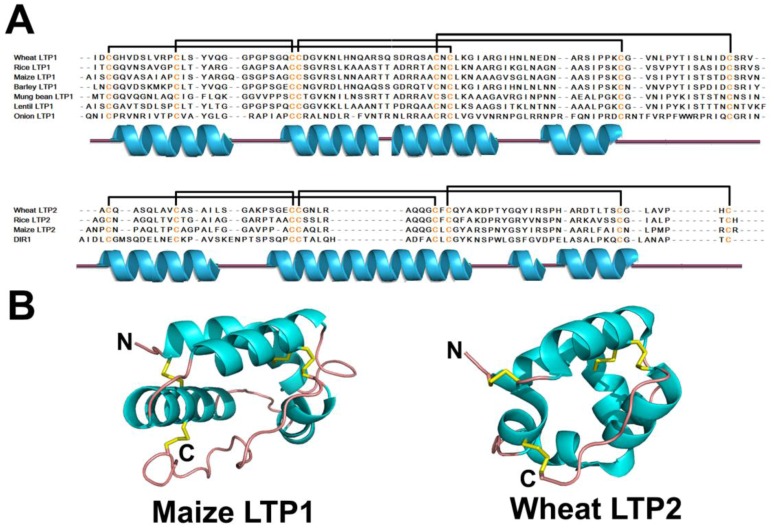
Sequences (**A**) and structures (**B**) of representative lipid transfer proteins. The secondary structure is represented by different colors: cyan-α helix; pink-random coil and yellow-disulfide bonds.

Although LTPs vary in their primary sequence, they share a defining structural feature, a conserved inner hydrophobic cavity surrounded by α-helices (Figure 8A,B). Surface plots of LTP1 and LTP2 representatives are illustrated in Figure 2G, H. LTP1s and LTP2s share the same Cys signature and similar tertiary fold, but vary in amino acid sequence and disulfide bonding at the CXC motif [248,249]. LTP1 contains four α-helices stabilized by four disulfide bonds (between CysI-CysVI, CysII-CysIII, CysIV-CysVII, and CysV-CysVIII) and a flexible C-terminal coil. In contrast to LTP1, LTP2 contains three extended helices, two single-turn helices, and four disulfide bonds between CysI-CysV, CysII-CysIII, CysIV-CysVII, and CysVI-CysVIII (Figure 8A) [249,250]. The helices in LTP1 and LTP2 form a hydrophobic cavity which accommodates a variety of lipids [251,252,253,254].

LTP1s and LTP2s have a conserved structure reported in rice [253,255,256], wheat [257,258], barley [259,260], maize [261,262], lentils [263], and mung beans [264]. Their lipid-binding properties can be modulated by subtle changes in their conserved global structure, as evidenced by structural and functional differences of LTP1s in tobacco, wheat, and maize [265]. Mutagenesis studies have revealed that different residues forming the hydrophobic cavity of LTP2 play various roles in maintaining structure and conferring function [266]. Although LTP2 is found to have a similar but smaller hydrophobic cavity compared to LTP1 [249,250,253,254,259], it is quite flexible and can accommodate voluminous sterol molecules [267].

#### 2.6.3. Structure-Function Relationship

Plant LTPs inhibit the growth of bacterial and fungal pathogens to different degrees [243,246,268]. Transgenic expression of barley LTP2 in tobacco and *Arabidopsis thaliana* leaves endows higher tolerance to bacterial pathogens [269]. The antifungal activity of LTPs from *Brassica* and mung beans is found to be thermally, pH-, and pepsin/trypsin treatment-stable [268].

Ace-AMP1 (onion LTP1; Figure 8A) isolated from onion seeds, exhibits a higher antimicrobial activity than the LTP extracted from radish seeds [89,243]. Ace-AMP1 shares a similar global fold as LTP1s, but does not possess a continuous cavity for lipid binding nor does it bind fluorescently labeled phospholipids in solution [252]. In addition, Ace-AMP1 does not exhibit characteristic LTP behavior, such as transferring lipids between membranes *in vitro*. While DIR1 from *Arabidopsis thaliana* shares similar structural and lipid binding properties with plant LTP2, it is distinguished by its acidic pI and ability to bind two long-chain fatty acid derivatives in its cavity, suggesting DIR1 could be a new type of plant LTP [270]. The functions of plant LTPs, such as defensive signaling, cuticle deposition and plant defense, have been previously reviewed by Yeats and Rose [271].

#### 2.6.4. Mechanism of Action

Initially, LTPs were reported to facilitate lipid transfer between membranes of vesicles or organelles *in vitro* [272,273]. However, later discoveries have shown that LTP1s are extracellular cell wall proteins, making *in vivo* intracellular lipid transfer activity unlikely [274,275]. Thus, LTPs promote membrane permeabilization in pathogens rather than host cells [246,276]. Although structural studies have shown that LTPs can “cage” lipid molecules in their hydrophobic cavity, a detailed mechanism of antimicrobial activity mediated by lipid transport remains unclear [271].

### 2.7. Snakins

Snakin-1 (63 aa) and snakin-2 (66 aa) are AMPs with 12 Cys isolated from potato tubers (*Solanum tuberosum*) [277,278] found to be active against fungal and bacterial pathogens at 1–20 μM. Snakins induce the aggregation of both Gram-positive and Gram-negative bacteria and therefore, are recognized as components of constitutive and inducible plant defense barriers. The structure of snakin is predicted to have two long α-helices with disulfide bonds between CysI-CysIX, CysII-CysVII, CysIII-CysIV, CysV-CysXI, CysVI-CysXII, and CysVIII-CysX, which demonstrates a small degree of structural similarity to thionins and α-hairpinins [279].

A 64 aa homolog of snakin-2, also containing 12 Cys, was identified from the French bean to be a domain of a 42 kDa Pro-rich protein with chitin-binding ability that is involved in plant-pathogen interactions [280]. Snakin-Z (31 aa), identified from the fruit of *Zizyphus jujuba,* contains a sequence similar to the C-terminal region of Snakin-2. It has antimicrobial activity against different bacterial and fungal strains at minimal concentrations (7.65–28.8 μg/mL) [281].

### 2.8. Other Plant CRP-AMPs

In addition to the different classes of CRP-AMPs mentioned above, there are several unclassified Cys-containing AMPs, such as Ps-AFP1, Ib-AMPs, Pp-AMPs, ToAMPs, and MiAMP1 that contain two to eight Cys. Ps-AFP1 (38 aa) from the pea *Pisum sativum* root contains two disulfide bonds between CysI-CysII and CysIII-CysIV, has been proposed to adopt a novel αβ-trumpet fold, and is capable of binding the cell wall of fungi [282]. Ib-AMPs1-4, purified from the seed of *Impatiens balsamina*, are four basic 20 aa peptides encoded by a single transcript. Ib-AMPs represent a novel class of AMPs that lack homology to other peptides [283,284]. They contain four Cys which share a common CC and CXXXC sequence motif and form two disulfide bonds between CysI-CysIII and CysII-CysIV. The structure of Ib-AMP1 is well-defined, with loops and turns stabilized by two disulfide bonds [285]. Ib-AMPs can inhibit the growth of a range of fungi and Gram-positive bacteria but is not cytotoxic to most Gram-negative bacteria or cultured human cells. Studies of Ib-AMP1 analogs without disulfide bonds have shown these bonds are not essential for its antimicrobial activity, and that Ib-AMP1 targets intracellular components instead of bacterial cell membranes [286].

Pp-AMP1 and Pp-AMP2 are two chitin-binding AMPs from the shoots of the Japanese bamboo *Phyllostachys pubescens* that have antimicrobial activity against pathogenic bacteria and fungi [287]. Pp-AMP1 contains four Cys in its 44 aa sequence, while Pp-AMP2 (45 aa) is composed of six Cys. Although they share relatively high sequence homology with thionins, especially the N-terminal continuous CC motif, their differences in the C-terminal sequence and Cys distribution pattern do not suggest Pp-AMPs are thionins. ToAMPs1-4 are four basic 38–44 aa AMPs identified from *Taraxacum officinale* flowers that have antifungal and antibacterial activity [288,289]. Sequence analysis has shown that ToAMP1, ToAMP2, and ToAMP4 possess a novel peptide motif of six Cys with a –CC– motif between CysII and CysIII, while ToAMP3 has eight Cys with a –CC– motif between CysVI and CysVII.

MiAMP1 is a 76 aa basic AMP identified from the nut kernel of *Macadamia integrifolia*. MiAMP1 inhibits several plant microbial pathogens *in vitro* [290], yet presents no sequence homology with other plant AMPs and has a β-barrelin structure. The β-barrelins contain eight β-strands forming two Greek key motifs that are stabilized by three disulfide bonds. MiAMP1 has a structure similar to that of a yeast killer toxin from *Williopsis mrakii*, which inhibits β-glucan synthesis and thereby disturbs the cell wall of yeast. The structural similarity between these two peptides suggests a similar antimicrobial mode of action.

### 2.9. Non-CRP Plant AMPs

Although the majority of known plant AMPs are stabilized by multiple disulfide bonds, exceptions include Cn-AMPs, Cr-ACP1, as well as GRPs Pg-AMP1 and shepherins. All of these plant AMPs either do not contain Cys or contain only one and thus, possess high structural flexibility. Cn-AMP1 is a 9 aa Cys-free AMP identified from green coconut (*Cocos nucifera*) water with “promiscuous” activity, as it is antibacterial, antifungal, and immune stimulatory [291]. Cr-ACP1 is a 9 aa AMP from the seeds of *Cycas revoluta* with pro-apoptotic and antimicrobial activities [292]. A previous bioinformatics study suggests that these Cr-AMP1 activities are likely mediated by hydrogen bond-mediated DNA binding within cells.

Pg-AMP1 is a 55 aa GRP extracted from seeds of the guava *Psidium guajava*. It contains 14 Gly and only one Cys [293] and inhibits the growth of both Gram-positive and Gram-negative bacteria, pathogens involved in urinary and gastro-intestinal infections [294]. Shepherins I and II are Gly- and His-rich AMPs identified from the roots of Shepherd’s Purse, *Capsella bursa-pastoris* [295]. Shepherin I (28 aa) and shepherin II (38 aa) are produced from a single 120 aa propeptide precursor composed of an N-terminal signal peptide, shepherin I, a linker dipeptide, shepherin II, and a C-terminal peptide. Both exhibit antimicrobial activity against Gram-negative bacteria and fungi with an IC_50_ of 2.5–8 μg/mL.

### 2.10. Mechanism of AMP Action

AMPs are generally moderate-to-large size, positively charged, amphipathic CRPs. Structurally, AMPs fall into diverse and distinct groups (Table 1; Figure 2), including α-helical peptides (α-hairpinin family and lipid transfer proteins), β-sheet peptides (hevein and knottin-type peptides) as well as mixed α-helical and β-sheet peptides (thionins and plant defensins). Generally, the mechanism of AMP interaction with microbes is believed to be associated with cell lysis due to membrane disruption and/or peptide penetration of lipid membranes followed by attack of intracellular targets [14]. Various mechanistic models have been proposed, such as the barrel-stave model, toroidal pore model, and carpet model, in previous reviews by Barbosa, Rahnamaeian, and Nawrot [14,296,297]. Besides perturbing the lipid membrane, AMPs can also form ion channels, which can induce ion leakage (e.g., K^+^) in addition to other intracellular contents [298,299]. All of these actions lead to inhibition of microbial cell growth and cell death.

## 3. Conclusions and Perspective

A striking feature of plant AMPs is that the majority are families of CRPs, with each family sharing a characteristic motif. In turn, these cysteinyl motifs enable plant AMPs to organize into specific families with conserved structural folds that enable sequence variation of non-Cys residues encased in the same scaffold within a particular family to play multiple functions. This evolvable phenomenon is particularly evident in the family of knottins, and to a lesser extent, in the defensins. Knottins are known to play diverse roles, being antimicrobial, insecticidal, enzyme inhibitory, and agonistic/antagonistic to hormones. The ability of plant AMPs to tolerate hypervariable sequences using a conserved scaffold mimic is, in certain respects, similar to that of immunoglobulins, which recognize diverse targets by varying the sequence of complementary binding regions.

The presence of multiple disulfide bonds in a particular CRP family gives plant AMPs a compact structure and a specific scaffold. In turn, they confer stability against thermal and chemical denaturation and enzymatic degradation. These properties bode well for developing plant AMPs as potential therapeutics and for protection of crops through transgenic methods [300,301]. A cystine-stabilized structure opens the possibility to use it as a stable scaffold for grafting biological active peptides sequences to intracellular targets. Specifically, the backbone portions between cysteine residues can be modified to incorporate bioactive peptides which are normally unstable against digestive enzymes and other physiological conditions [302,303]. This technique has successfully been applied to graft bradykinin B1 antagonists onto kalata B1 as an orally active analgesic [221], human kallikrein-related peptidase 4 inhibitor onto sunflower trypsin inhibitor-1 [228], angiogenic peptides, laminin and osteopontin, onto *Momordica cochinca cochinchinensis* trypsin inhibitor-II [304] and melanocortin onto kalata B1 [305]. More importantly, these grafted peptides have been shown to possess the same biological property with similar potency as compared with the original bioactive peptides, as well as resistance to digestive enzymes due to the robust scaffold. In addition, plants AMPs, in particular defensin, have been shown to protect transgenic plants against microorganism infection. Expression of alfalfa antifungal peptide defensin, from the seeds of *Medicago sativa*, in transgenic potato plants has been found to provide resistance against phyto-pathogen *Verticillium dahilae* [77,306].

AMPs offer several advantages as compared to current antibiotic drugs as they represent a naturally occurring defense mechanism that has been used by plants for thousands of years in combating external pathogenic challenges. Most of the recent studies included in this review were focused on the anti-fungal and anti-bacterial effect of AMPs, while the anti-viral property of AMPs was still under-explored. Further investigation in tackling this issue is urgently warranted. Since plant AMPs have been identified in only a small fraction of plants, it is anticipated that many more plant AMPs, both in molecular form and sequence, will be forthcoming.
